# Determinants of Hypoglycemia in Premature Vietnamese Infants: A Case-Control Study

**DOI:** 10.7759/cureus.52905

**Published:** 2024-01-25

**Authors:** Rang N Nguyen, Tuong M Tran, Ly Lien H Le, Chanh Q Ton

**Affiliations:** 1 Department of Pediatrics, Can Tho University of Medicine and Pharmacy, Can Tho, VNM; 2 Department of Pediatrics, The Women and Children Hospital of An Giang, An Giang, VNM

**Keywords:** premature infants, gestational hypertension, gestational weight gain, gestational diabetes, neonatal hypoglycemia

## Abstract

Background

Premature infants are more likely to experience hypoglycemia. Early recognition and prompt therapy are essential to avoiding neurological sequelae in the future. This study aimed to identify the determinants of hypoglycemia in premature Vietnamese infants.

Methodology

This was a case-control study conducted at the Neonatal Intensive Care Unit, The Women and Children Hospital of An Giang, Vietnam. Hypoglycemia was defined as a plasma glucose value of less than 2.6 mmol/L (47 mg/dL) after two hours postpartum. Maternal and neonatal information was collected and analyzed. Both bivariate and multiple logistic regression models were used to identify the risk factors of neonatal hypoglycemia (NH)

Results

A total of 65 cases and 195 controls were included in the study. Gestational diabetes mellitus (GDM) (adjusted odds ratio [AOR] 3.78, 95% confidence interval [CI] 1.69-8.52; *P *< 0.001) and excessive gestational weight gain (GWG) (AOR 2.80, 95% CI 1.12-6.98; *P* < 0.026) were associated with NH in the multiple logistic regression model. An observed positive interaction between gestational hypertension and GDM on NH yielded an odds ratio (OR) of 6.29 (95% CI 2.46-16.64).

Conclusions

GDM, excessive GWG, and the interaction between gestational hypertension and GDM were the determinants of hypoglycemia in premature infants.

## Introduction

Hypoglycemia is the most common metabolic disturbance occurring in the neonatal period. Neonatal hypoglycemia (NH) has an overall incidence of 1-5 per 1,000 live births, with a higher incidence observed in premature infants (16%-38%) [1-4]. Early identification and prompt treatment of NH are of great importance, since hypoglycemia in the neonatal period may lead to neurodevelopmental sequelae [5].

Neonates at risk of NH include infants born to mothers with gestational diabetes mellitus (GDM), large for gestational age (LGA), small for gestational age (SGA), and premature neonates (<37 weeks gestation) [6,7]. Furthermore, several risk factors for NH have been reported in previous studies in different countries, including gestational hypertension (GH), preeclampsia and eclampsia during pregnancy, maternal intravenous glucose infusion [8,9], and antenatal steroid administration [10]. Recently, some studies suggested that overweight, or obesity, and excessive gestational weight gain (GWG) may also lead to NH [11-13]. Although overweight/obesity is not as prevalent in Vietnam as it is in developed countries, excessive GWG may also result in NH in our population.

In addition to maternal risk factors, cesarean section delivery and various clinical conditions of neonates may lead to hypoglycemia, including asphyxia, hypothermia, polycythemia, and sepsis [14-16].

To date, a few studies on NH in Vietnam have been reported. This study aims to identify maternal or neonatal determinants of hypoglycemia in premature Vietnamese neonates, focusing on the role of excessive GWG in NH.

## Materials and methods

Study design and setting

It is a prospective case-control study. Premature neonates with hypoglycemia were considered cases. Each case was matched with three consecutive control cases of preterm neonates without hypoglycemia. This study was conducted at the Neonatal Intensive Care Unit (NICU) of The Women and Children Hospital of An Giang, Vietnam, between January 2022 and June 2023. All premature neonates under two days of age were included. Neonates with major congenital malformations and those born to mothers with type 1 diabetes mellitus were excluded from this study. 

Sample size

With power at 80%, confidence level at 95%, a ratio of control to a case of 3:1, an odds ratio (OR) of 2.46, and an expected proportion exposed in controls of 16.7% [9], 65 cases and 195 controls were calculated as the final sample size. StatCalc of Epi Info version 7 was used to calculate the sample size.

Data collection

We collected data concerning mothers and infants by directly interviewing the mothers, reviewing maternal medical records to add some missing data, and clinical examinations of infants. All mothers were interviewed by trained nurses, and all infants were examined by attending physicians. The whole data collection process was controlled and supervised by the project manager.

The main mother-related parameters were age, height, body weight before pregnancy and at birth, parity, history of GDM, GH, antenatal steroids administration, and glucose intravenous perfusion.

The main infant-related parameters were gender, gestational age, birth weight, delivery mode, and clinical conditions that may lead to hypoglycemia (asphyxia, hypothermia, polycythemia, and sepsis).

Operational Definitions

- NH was defined as a plasma glucose value of less than 2.6 mmol/L (47 mg/dL) after two hours postpartum as confirmed by using a glucose oxidase method [1].

- Preterm birth was defined as birth before 37 weeks of pregnancy. Gestational age was determined by the date of the last menstrual cycle and corrected by ultrasound as necessary.

- Infants were determined to be appropriate for gestational age (AGA) if their birth weight was between the 10th and 90th percentiles for their gestational age and sex. Infants were determined to be SGA or LGA if their birthweight was less than the 10th percentile or greater than the 90th percentile for their gestational age and sex, respectively.

- Pre-pregnancy body mass index (BMI; weight in kg/[height in m]^2^) was categorized into three groups: underweight (<18.5 kg/m^2^), normal weight (18.5-24.9 kg/m^2^), and overweight/obese (≥25.0 kg/m^2^). Excessive GWG was defined as a weight gain of more than 18 kg in underweight, >16 kg in normal weight, and >11.5 kg in overweight women [17].

- GDM was diagnosed as follows: fasting plasma glucose level (PG) ≥ 92 mg/dL (5.1 mmol/L); one-hour PG ≥ 180 mg/dL (10.0 mmol/L); two-hour PG ≥ 155 mg/dL (8.5 mmol/L) using the 75-g glucose tolerance test [18].

- GH was defined as blood pressure greater than or equal to 140 mmHg systolic or 90 mmHg diastolic after 20 weeks of pregnancy [19].

- Regarding clinical conditions of neonates: Hypothermia was defined as a body temperature less than 36.5 °C; polycythemia was defined as a hematocrit level from peripheral venous blood greater than or equal to 65%; asphyxia was defined as an APGAR score less than or equal to 7 at five minutes; and neonatal sepsis was diagnosed based on clinical symptoms and a positive blood culture [20].

Ethical issues

This research protocol was approved by the Ethics Committee of The Women and Children Hospital of An Giang (approval no. 35c-QĐBVSN on November 30, 2021). Each mother provided written consent before the interview was conducted.

Statistical analysis

Continuous variables are described using mean with standard deviation (SD) for normally distributed continuous variables. Categorical variables were described using frequencies and percentages. A chi-square test was used for categorical variables, or Fisher's exact test was utilized when the expected values in any of the cells were below 5. Univariate logistic regression models were conducted to identify variables significantly associated with the outcome (NH). We checked multicollinearity and examined for any interaction between independent variables in association with dependent variables. The potential factors associated with the dependent variable were applied to multivariate logistic regression analysis. Results were presented as odds ratio (OR) and 95% confidence intervals (CIs). Analyses were performed using R version 4.3.0 (https://cran.r-project.org/). All tests of statistical significance were two-tailed, and *P* < 0.05 was considered statistically significant.

## Results

Neonatal characteristics of cases and controls

The study included and analyzed 270 neonates (65 cases and 195 controls) admitted to the NICU. The mean gestational age (±SD) between cases and controls was 33.2 ± 2.5 and 33.0 ± 2.9 weeks (*P *= 0.579), respectively. The mean birth weight (±SD) between cases and controls were 1893 ± 533 and 1891 ± 526 g (*P *= 0.989), respectively.

The majority of the cases 34 (72.8%) and the controls 112 (76.7%) were females. A higher proportion of neonates born LGA was observed in cases (33, 50.8%) compared to controls (72, 36.9%). The proportion of neonates born SGA in cases (7, 10.7%) was more prevalent than in controls (9, 4.6%). The mode of delivery and the parity were not different between cases and controls. Clinical conditions at risk of NH (asphyxia, hypothermia, polycythemia, and sepsis) were not different between cases and controls (Table 1).

**Table 1:** The comparisons of neonatal characteristics between the hypoglycemia group and the nonhypoglycemia group. GA/BW, gestational age and birth weight; AGA, appropriate for gestational age; SGA, small for gestational age; LGA, large for gestational age

Variables	Hypoglycemia (*n* = 65), *n* (%)	Nonhypoglycemia (*n* = 195), *n* (%)	*P*-value
Gender			
Male	31 (27.2)	83 (23.3)	
Female	34 (72.8)	112 (76.7)	0.471
GA/BW classification			
AGA	25 (38.5)	114 (58.5)	
SGA	33 (50.8)	72 (36.9)	
LGA	7 (19.7)	9 (4.6)	0.011
Mode of delivery			
Vaginal	36 (55.4)	116 (59.5)	
Cesarean	29 (44.6)	79 (40.5)	0.561
Clinical conditions			
Asphyxia			
Yes	1 (1.5)	5 (2.6)	
No	64 (98.5)	190 (97.4)	0.633
Hypothermia			
Yes	1 (1.5)	2 (1.0)	
No	64 (98.5)	193 (99.0)	0.412
Polycythemia			
Yes	4 (6.2)	11 (5.6)	
No	61 (93.8)	184 (94.4)	0.737
Sepsis			
Yes	18 (27.7)	50 (25.6)	
No	47 (72.3)	145 (74.4)	0.746

Maternal characteristics of cases and controls

The majority of mothers in cases and controls were less than 35 years of age (50, 76.9%; 142, 72.8%). Overweight (BMI > 25 kg/m^2^) was more prevalent in cases (15, 23.1%) than in controls (17, 8.7%) (*P *= 0.007). The proportion of mothers with excessive GWG was higher in cases (19, 29.2%) than in controls (18, 9.2%) (*P *< 0.001). Mothers with GDM were more commonly identified in cases (19, 29.2%) than in controls (18, 9.2%) (*P *< 0.001), and mothers with GH was also more prevalent in cases (21, 32.3%) than in controls (35, 17.9%) (*P *< 0.001).

Nearly one-third (20, 30.8%) of mothers in cases and controls (58, 29.7%) received antenatal steroids, and one-fifth (13, 20%) of mothers had intravenous glucose infusion compared to 53 (27.2%) in controls (Table 2).

**Table 2:** The comparisons of maternal characteristics between the hypoglycemia group and the non-hypoglycemia group ^*^P-value from chi-square test. GWG, gestational weight gain; GDM, gestational diabetes mellitus; GH, gestational hypertension; IV, intravenous; BMI, body mass index

Variables	Hypoglycemia (*n* = 65), *n* (%)	Nonhypoglycemia (*n* = 195), *n* (%)	*P*-value
Mother age (years)			
<35	50 (76.9)	142 (72.8)	
≥35	15 (23.1)	53 (27.2)	0.499
BMI (kg/m^2^)			
<18.5	4 (6.1)	21 (10.8)	
18.5-24.9	46 (71.8)	157 (80.5)	
≥25	15 (23.1)	17 (8.7)	0.007
Parity			
Nulliparous	34 (52.3)	111 (56.9)	
Multiparous	31 (47.7)	84 (43.1)	0.516
Excessive GWG			
Yes	19 (29.2)	18 (9.2)	
No	46 (70.8)	177 (90.8)	<0.001
GDM			
Yes	19 (29.2)	18 (9.2)	
No	46 (70.8)	177 (90.8)	<0.001
GH			
Yes	21 (32.3)	35 (17.9)	
No	44 (67.7)	160 (82.1)	<0.001
Antenatal steroids			
Yes	20 (30.8)	58 (29.7)	
No	45 (69.2)	137 (70.3)	0.015
IV glucose infusion			
Yes	13 (20.0)	53 (27.2)	
No	52 (80.0)	142 (72.8)	0.876

Determinants of NH among premature infants

In univariate logistic regression analysis, infants with SGA and LGA, maternal overweight, excessive GWG, GH, and GDM were statistically significantly associated with NH (Table 2). 

In multivariate logistic regression analysis, mothers with excessive GWG and GDM were both determinants of NH, with an adjusted odds ratio (AOR) of 2.80 (95% CI 1.12-6.98; *P *= 0.026) and 3.78 (95% CI 1.69-8.52; *P *= 0.001), respectively (Table 3).

**Table 3:** Factors associated with hypoglycemia in univariate and multivariate logistic regression analyses. COR, crude odds ratio; AOR, adjusted odds ratio; CI, confidence interval; AGA, appropriate for gestational age; SGA, small for gestational age; LGA, large for gestational age; GWG, gestational weight gain; GH, gestational hypertension; GDM, gestational diabetes mellitus

Variables	COR (95% CI)	*P*-value	AOR (95% CI)	*P*-value
Gender				
Female	1		1	
Male	0.81 (0.46-1.43)	0.047	0.69 (0.36-1.31)	0.259
Gestational age				
ASA	1		1	
SGA	2.09 (1.15-3.82)	0.015	1.75 (0.90-3.40)	0.096
LGA	3.54 (1.16-10.44	0.021	3.12 (0.90- 10.3)	0.063
Maternal BMI				
Normal	1		1	
Underweight	0.50 (0.11-1.55)	0.285	0.63 (0.14-2.02)	0.486
Overweight/obesity	2.96 (1.36-6.40)	0.005	1.59 (0.58-4.16)	0.347
Excessive GWG				
No	1		1	
Yes	4.06 (1.97-8.42)	<0.001	2.80 (1.12-6.98)	0.026
GH				
No	1		1	
Yes	2.18 (1.14-4.10)	0.027	1.21 (0.58-2.45)	0.605
GDM				
No	1		1	
Yes	4.06 (1.97-8.42)	<0.001	3.78 (1.69-8.52)	0.001

It was found that GH and GDM had a significant interaction effect on the outcome variable (NH) (*P*_interaction_ = 0.05). It was shown that there was an additive interaction between GH and GDM in NH. With the interaction term, the OR of developing NH increased from 4.06 (CI 95% 1.97-8.42) to 6.29 (CI 95% 2.46-16.64) (Table 4).

**Table 4:** The interaction between GH and GDM on NH. GH, gestational hypertension; GDM, gestational diabetes mellitus; OR, odds ratio; CI, confidential interval; NH, neonatal hypoglycemia

GH	GDM	Estimate coefficients	OR	95% CI
1	0	0,780	2.18	1.14-4.10
0	1	1.401	4.06	1.97-8.42
1	1	1.839	6.29	2.46-16.64

## Discussion

This is the first case-control study examining hypoglycemia in premature infants in Vietnam. This study aims to re-evaluate the determinants of NH from previous reports in different countries. A multivariate logistic regression analysis of this study identified excessive GWG, GDM, and GH as additive interactions with GDM, which are risk factors for hypoglycemia in premature Vietnamese infants.

According to this study, neonates born to women with excessive GWG had about three times higher odds of having NH than those born to women without excessive GWG. The results of our study were similar to those of three studies conducted in the United States. In a nested case-control study of 45,245 women with singleton delivery in California, Hedderson et al. found that women with excessive GWG were associated with the risk of NH (OR = 1.38, 95% CI 1.01-1.89) [21]. A cohort of 29,861 delivered women in 25 different hospitals demonstrated that neonates born to women with excessive GWG were 60% more likely to develop NH (OR = 1.6, CI 95% 1-4.2) [22]. In a retrospective cohort of 23,902 term infants, Erin et al. found that women with excessive GWG had significantly higher odds of NH (AOR = 1.15; *P *= 0.01) [12]. This might be explained by the fact that excessive GWG in the second half of pregnancy affects glucose metabolism. Maternal hyperglycemia due to decreased insulin sensitivity leads to high levels of blood glucose and hyperinsulinemia in a fetus. NH occurs when the blood supply from mothers ceases immediately after delivery [23-24].

According to our study, premature neonates born to mothers with GDM had higher odds of NH than those born to mothers without GDM. Our study is consistent with previous studies in other countries. An analysis of 45,245 women conducted in the United States found that women with GDM were more likely to have infants with hypoglycemia (OR = 2.61, 95% CI 0.99-6.92) [25]. According to a Chinese study of 1,253 women with GDM, diabetic women with excessive GWG are more inclined to develop NH than those without excessive GWG (OR = 3.8, 95% CI 1.22-5.3) [26]. Researchers in Australia found that 47% of infants born to diabetic mothers had hypoglycemia, increasing their risk of developing NH (OR = 7.2, CI 95% 1.3-40.7) [27]. It is suggested that pregnant women with GDM may not produce enough insulin to overcome the increased insulin resistance caused by hormonal changes during pregnancy. Therefore, maternal hyperglycemia leads to excess glucose exposure in the fetus and subsequent fetal hyperinsulinemia, resulting in NH postpartum [ 28]

GH has been associated with NH in previous studies. Performing a study on 515 infants born less than 32 weeks gestation in China, Yuan et al. found that maternal hypertension was a risk factor for NH (OR = 2.469, 95% CI 1.310-4.652; *P *< 0.05) [9]. Similarly, Mitchell et al. found that maternal hypertension was the only risk factor for hypoglycemia in premature infants born at 33 weeks gestation (OR = 3.07, 95% CI 1.51-6.30; *P *= 0.002) [3]. In our study, maternal hypertension was not an independent risk factor for hypoglycemia in premature neonates, but it interacts with GDM to develop NH. Our study found there was a 1.5-fold increase in NH among diabetic mothers with GH compared to diabetic mothers without GH. This may be because the presence of GH and GDM in the mother during pregnancy can cause placental dysfunction, reducing the transfer of nutrients to the fetus from the mother. A compromised placenta can reduce glucose levels in the neonate, increasing the risk of hypoglycemia [29].

In previous studies, it has been reported that cesarean section increases the risk of hypoglycemia in neonates, possibly due to delayed breastfeeding [9,14,30]. However, cesarean section was not associated with NH in our study.

SGA infants were not at increased risk for NH in this population; however, previous studies have demonstrated that these neonates are at risk for hypoglycemia [5,9]. Our study also found no association between LGA infants and NH, consistent with prior studies in developed countries [3,14].

In our study population, antenatal steroid administration was neither identified as a significant risk factor nor shown to be protective. In contrast, Dude et al. found that corticosteroid administration is associated with higher odds of NH (AOR = 2.96, 95% CI 1.29-6.82); however, all mothers in the study of Dude et al. had diabetes mellitus [10].

Researchers in developing countries have identified that infants with asphyxia, hypothermia, or neonatal sepsis are at risk of NH [8,30], but we did not find a statistically significant association between these diseases and NH since the incidence of these diseases was low in our population.

One of the study's strengths was that data collected from mothers were reliable since most women and their children received antenatal care and delivered in our hospital. The limitations of the study were as follows: (1) case-control studies were prone to bias because of their nature; moreover, due to their small size, all potential confounders could not be controlled. (2) The results of this study could not be generalized since it was conducted only at one institution. (3) Measurement of blood insulin levels was not possible, and congenital diseases such as hyperinsulinism and hypopituitarism were not identified among newborns.

## Conclusions

NH is a common pathology in premature infants in our hospital. Mothers with gestational diabetes, as well as the interaction between GH and gestational diabetes, are determinants for NH. It was worth noting that excessive GWG is a distinctive predictor of developing NH in our population, where maternal overweight/obesity is not as prevalent as in Western countries. However, a larger sample size is needed to identify excessive GWG as a unique predictor of NH in developing countries.
